# Atherosclerosis: From Molecular Biology to Therapeutic Perspective 2.0

**DOI:** 10.3390/ijms232315158

**Published:** 2022-12-02

**Authors:** Ida Perrotta

**Affiliations:** Department of Biology, Ecology and Earth Sciences, Centre for Microscopy and Microanalysis (CM2), University of Calabria, Arcavacata di Rende, 87036 Cosenza, Italy; idaperrotta@yahoo.it

Atherosclerosis is a chronic inflammatory disease of large- and medium-sized arteries involving aberrant immune–inflammatory responses, dysfunctional molecular pathways, and impaired tissue repair mechanisms [1]. Atherosclerosis begins with endothelial damage, proceeds with the subendothelial retention of modified low-density lipoproteins (LDL) and the phenotypic switching of the smooth muscle cells (SMCs), and culminates with plaque instability and eventually with plaque rupture with its often-fatal sequelae [2,3,4]. Over recent years, the rapidly evolving field of cellular and molecular biology has improved our ability to characterize the lesions beyond their anatomical structure, revealing new insights into the signaling axes that typify vascular behavior and regulate multiple critical steps in the pathogenesis of atherosclerosis. These include previously unknown biochemical signals and molecular pathways whose activities and interactions with their surrounding environment could contribute to atherosclerosis by initiating the lesion and participating in its progression. There has also been an important recognition of the role of exosomes and their miRNAs in different processes that appear to significantly contribute to the pathophysiology of the disease [5]. Substantial progress continues to be made toward understanding the complex interplay between various genetic, epigenetic, and environmental risk factors implicated in the pathogenesis of atherosclerosis [6,7]. Finally, there have been some exciting discoveries in the therapeutic arena with tangible improvements in patient outcomes.

This Special Issue contains research articles and reviews written by experts in the field and focused on the cellular and molecular mechanisms of atherosclerosis and their pivotal roles in the development and progression of the plaque. This Special Issue also explores the classic and more recently defined clinical risk factors for atherosclerotic cardiovascular disease, outlines new promising approaches for its early detection and treatment, and highlights areas where more research activities could usefully be directed.

Vascular SMCs are key participants in both early atherogenesis and advanced plaque progression. SMCs are not terminally differentiated and can modulate their phenotype in response to environmental cues, such as growth factors, inflammatory mediators, mechanical forces, cell–cell and cell–matrix interactions, extracellular lipids, and lipoproteins. Although SMCs can undergo multiple phenotypic transitions, a hallmark feature of SMC phenotypic switching during atherosclerosis is the downregulation/loss of markers and pathways associated with SMC differentiation that facilitate their capability to migrate into the intima and proliferate, thereby contributing to neointimal lesions [8]. As such, there has been significant interest in identifying the molecules and mechanisms involved in suppressing SMC differentiation marker gene expression. Chiu et al. demonstrated that Yohimbine (YOH), a phytochemical with strong anti-proliferative effects against breast and pancreatic cancer cells, can attenuate the proliferation and migration of vascular SMCs in vivo through the downregulation of the α2B-adrenergic receptor-independent PLCγ1 pathway. Additionally, the paper of Roberts-Craig and colleagues carefully reviewed existing knowledge regarding the role of CaMKII within vascular SMCs and the molecular mechanisms that regulate its activity and highlighted the need for further research to test its cardiovascular effects. Based on the recent discovery that smooth muscle progenitors contribute to the development of atherosclerotic lesions, Balatskiy et al. demonstrated that despite immature SMCs often existing in areas that are prone to atherosclerotic disease and their number increasing with age, their migratory ability and immunophenotypic profile do not support a pro-atherogenic role for this subpopulation of SMC in the arterial intima.

Alongside their obvious function in primary hemostasis and thrombosis, platelets can also play important roles during inflammation, especially in vascular diseases. Evidence suggests that platelets can interact with white blood cells and vascular endothelial cells both in a contact-dependent manner and/or through the release of various immune mediators [9]. Dziedzic et al. investigated the molecular mechanisms involved in the interactions between blood platelets and leukocytes in atherosclerotic patients with non-fatal acute coronary syndrome (ACS) complications and without typical risk factors. Their results demonstrated the presence of a significant alteration in the expression levels of various inflammatory mediators derived from the peripheral blood cells that propagate and mature impaired immune responses and systemic inflammation that, in turn, exert a detrimental influence on the pathogenesis of ACS. The role of platelets in atherosclerosis has also been carefully analyzed by Guan and colleagues that demonstrated the pro-thrombotic activity and the prognostic significance of the platelet CXCL16–CXCR6 axis in patients with ACS.

Hypertension, diabetes mellitus (DM), and hypercholesterolemia represent the most important risk factors responsible for the high prevalence and incidence of atherosclerosis [10]. Boarescu et al. demonstrated that all these risk factors significantly contribute to the development of oxidative stress, elevating the tissue levels of various pro-inflammatory cytokines and producing irreversible pro-atherogenic changes in the vessel wall that increase arterial stiffness and decrease its elasticity. Currently, most pharmacological and non-pharmacological treatment approaches aim to control the major and modifiable risk factors for atherosclerosis. Hyperglycemia, associated with uncontrolled DM, is closely related to cardiovascular diseases. In their work, Janjusevic and co-workers provided interesting details about the latest advancements regarding antidiabetic and antiplatelet therapies coupled with vitamin D supplementation to reduce blood glucose. Advanced glycation end products (AGEs) constitute a diverse group of compounds that become glycated after exposure to sugars whose potentially harmful effects depend on their ability to promote oxidative stress, inflammation, and apoptosis. AGEs can also activate specific receptors on the surface of vascular cells, leading to the activation of signaling pathways relevant to atherosclerotic processes. The review by Pinto et al. discussed the main mechanisms of action of AGEs and their interaction with their receptors with a specific focus on the pathways controlling cholesterol accumulation, oxidative stress, inflammation, and endoplasmic reticulum stress in the arterial wall.

Another important event contributing to the development of atherosclerosis is vascular inflammation, with immune-competent cells in lesions (predominantly activated macrophages) producing pro-inflammatory cytokines. The review by Barbu and co-workers accurately reviewed the association between inflammation and diabetes, obesity, premature vascular aging, and atherothrombosis, while Sagris et al. reported on the relationship between inflammation and coronary microvascular dysfunction (CMVD) and possible therapeutic strategies. This review also introduces the research progress regarding developing a novel, non-invasive approach to assess CMVD.

An interesting contribution of calciprotein particles (CPPs) to the pathogenesis of atherosclerosis has been proposed by Shishkova and co-workers. CPPs are colloidal nanoparticles dispersed in the human blood upon its supersaturation with calcium and phosphate. The results presented in this Special Issue demonstrate that patients with coronary artery disease or cerebrovascular disease show a predisposition to developing CPPs and that intravenous infusion of CPPs in normotensive and normolipidemic Wistar rats can induce adventitial inflammation and intimal hyperplasia in both intact and balloon-injured arteries in the absence of other CVD risk factors. In vitro studies also indicate that the exposure of primary human arterial endothelial cells to CPPs results in the secretion of pro-inflammatory cytokines, conversion of endothelial-to-mesenchymal cells, adhesion of leukocytes to vascular endothelium, and lysosomal cell death. Based on these findings, the authors conclude that CPPs can potentially lead to the development of cardiovascular disease through the induction of inflammatory processes and endothelial dysfunction.

As briefly mentioned above, atherosclerotic lesions are promoted by low-density lipoproteins that initiate a cascade of multiple signaling events responsible for the functional and structural alterations of the vessel wall. This Special Issue contains two reviews that provide insight into the role of lipids and lipoproteins in cardiovascular disease. The first review by Kotlyarov et al. describes the dynamics of long-chain fatty acids and their metabolites in the inflammatory responses that lead to the development of atherosclerotic plaque with emphasis on the role of free fatty acids in endothelial dysfunction; the second one by Yanai et al. discusses different atherogenic factors, including lipoprotein remnants, Lipoprotein(a), malondialdehyde-modified LDL (MDA-LDL), and small-dense LDL (Sd-LDL), that may contribute to residual CV risk in patients who are already being treated with statins.

Two separate reviews focus on emerging therapeutic strategies to better treat CVDs. Heo and Kang report on the effects of exosomes derived from different tissue sources that can elicit in atherosclerotic diseases, while Dabravolski et al. emphasize the role of HSP90 protein in cardioprotection and its association with atherosclerosis and heart aging.

I would like to express my special appreciation and thanks to all editorial board members, reviewers, and authors, experts in the field of cardiovascular research, without whose cooperation the present Special Issue would not have come into being.

I sincerely hope that you will enjoy reading this Special Issue and that it will stimulate new ideas for improvement. Happy reading!

## References

[1] Singh R.B., Mengi S.A., Xu Y.J., Arneja A.S., Dhalla N.S. (2002). Pathogenesis of atherosclerosis: A multifactorial process. Exp. Clin. Cardiol..

[2] Davignon J., Ganz P. (2004). Role of endothelial dysfunction in atherosclerosis. Circulation.

[3] Grootaert M.O.J., Martin R Bennett M.R. (2021). Vascular smooth muscle cells in atherosclerosis: Time for a re-assessment. Cardiovasc. Res..

[4] Koenig W., Khuseyinova N. (2007). Biomarkers of Atherosclerotic Plaque Instability and Rupture. Arterioscler. Thromb. Vasc. Biol..

[5] Wang C., Li Z., Liu Y., Yuan L. (2021). Exosomes in atherosclerosis: Performers, bystanders, biomarkers, and therapeutic targets. Theranostics.

[6] Tang H., Zeng Z., Shang C., Li Q., Liu J. (2021). Epigenetic Regulation in Pathology of Atherosclerosis: A Novel Perspective. Front. Genet..

[7] Reschen M.E., Lin D., Chalisey A., Soilleux E.J., O’Callaghan C.A. (2016). Genetic and environmental risk factors for atherosclerosis regulate transcription of phosphatase and actin regulating gene PHACTR1. Atherosclerosis.

[8] Allahverdian S., Chaabane C., Boukais K., Francis G.A., Bochaton-Piallat M.L. (2018). Smooth muscle cell fate and plasticity in atherosclerosis. Cardiovasc. Res..

[9] Morrell C.N., Aggrey A.A., Chapman L.M., Modjeski K.L. (2014). Emerging roles for platelets as immune and inflammatory cells. Blood.

[10] Petrie J.R., Guzik T.J., Touyz R.M. (2018). Diabetes, Hypertension, and Cardiovascular Disease: Clinical Insights and Vascular Mechanisms. Can. J. Cardiol..

